# Supplementary material for: Development and validation of prediction models for predicting social care strengths and vulnerability in older people: Cohort study using routine data in Adult Social Care
Source: PLoS One. 2026 Apr 15;21(4):e0328330. doi: 10.1371/journal.pone.0328330 (PMC13082613; doi:10.1371/journal.pone.0328330)
Supplement: S1 Table — (PDF) [file pone.0328330.s002.pdf]

**Table S1. Missingness in variables used in the logistic models n=20,218**

| Variable Name | % Missing (rounded to the nearest one decimal point) |
|---|---|
| Age | 0 |
| Sex | 0 |
| Ethnicity | 0 |
| IMD | 6.6 |
| Living Situation | 69.8 |
| Tenure | 38.8 |
| ADL Eat | 76 |
| ADL Personal Hygiene | 76 |
| ADL Toilet | 76 |
| ADL Clothed | 76 |
| ADL Home | 76 |
| Carer Access | 0 |
| Primary Support Reason | 63.7 |
| Cognitive Impairment | 0 |
| Remains at Home | 0 |
