# Supplementary figures and images for: Development and validation of prediction models for predicting social care strengths and vulnerability in older people: Cohort study using routine data in Adult Social Care
Source: PLoS One. 2026 Apr 15;21(4):e0328330. doi: 10.1371/journal.pone.0328330 (PMC13082613; doi:10.1371/journal.pone.0328330)

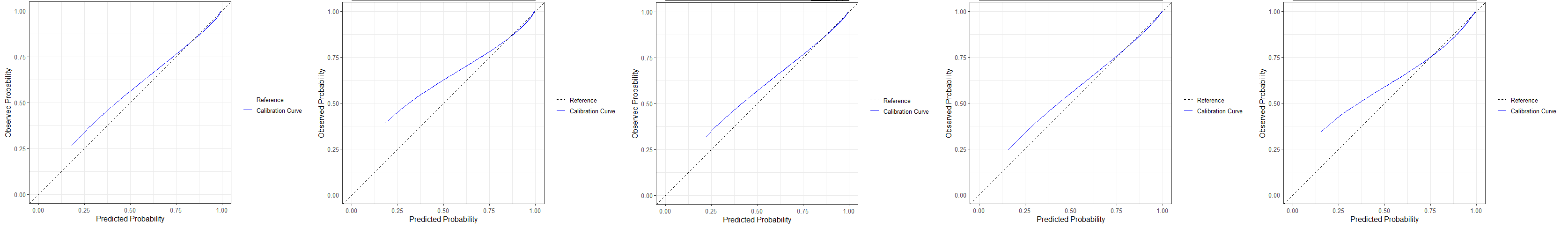

Supplement: S1 Fig — (PNG) [file pone.0328330.s003.png]
