# Supplementary material for: Development and validation of prediction models for predicting social care strengths and vulnerability in older people: Cohort study using routine data in Adult Social Care
Source: PLoS One. 2026 Apr 15;21(4):e0328330. doi: 10.1371/journal.pone.0328330 (PMC13082613; doi:10.1371/journal.pone.0328330)
Supplement: S2 Fig — (PDF) [file pone.0328330.s004.pdf]

Example of how to use the tool to identify a relatively high-risk case

Imagine a hypothetical case of a 70-year woman from a relatively deprived area of the inner city (IMD=46) living in a private tenancy, with ‘other’ (non-white) ethnicity. In terms of need, they would have been assessed as not able to feed themselves or look after their home, but were able to bathe and groom, use the toilet and dress themselves. Their records would show that they did not have access to a carer and their Primary Support Reason for accessing social care would be for ‘mental health support’. They would be known to have a dementia diagnosis.

The Figure shows how one could make a prediction for such a hypothetical person, who would have entered the social care system, with these items of information available in their record.

The local authority team manager or social worker would input this information in the tool on the left-hand side check boxes. This would generate the risk result on the right-hand side.

For this hypothetical case, there would be a **12% probability** of them being admitted to a care home, two years after their initial social care assessment.

VisABLE Risk Calculator

Age: (person's age in years)

70

IMD: (the index of multiple deprivation value for where the person lives)

46

Sex: (person's biological sex)

- (●) Female
- ( ) Male
- ( ) Refused/Missing

Ethnicity: (ethnicity based on simple catagories)

- ( ) White
- ( ) Mixed
- ( ) Asian or Asian British
- ( ) Black or Black British
- (●) Other
- ( ) Refused
- ( ) Missing

Tenure: (type of accommodation person occupies)

- ( ) Owner Occupier
- (●) Private Tenant
- ( ) Housing Association
- ( ) Council
- ( ) Sheltered Housing
- ( ) Residential Care
- ( ) Other

ADLeat: (is the person able to feed themselves as an activity of daily living)

- (●) No
- ( ) Yes
- ( ) Not Available

ADLHyg: (is the person able to bathe and groom themselves as an activity of daily living)

- ( ) No
- (●) Yes
- ( ) Not Available

ADLToilet:(is the person able to use the toilet by themselves as an activity of daily living)

- ( ) No
- (●) Yes
- ( ) Not Available

ADLDressing: (is the person able to dress themselves as an activity of daily living)

- ( ) No
- (●) Yes
- ( ) Not Available

ADLHome: (is the person able to look after themselves as an activity of daily living)

- (●) No
- ( ) Yes
- ( ) Not Available

CarerAccess: (whether person has access to a carer)

- (●) No
- ( ) Yes
- ( ) Not Available

Primary Support: (the primary reason the person is being referred or supported by social care)

- ( ) Access & Mobility
- (●) Mental Health
- ( ) Memory & Cognition
- ( ) Visual Impairment
- ( ) Hearing Impairment
- ( ) Dual Impairment
- ( ) Learning Disability
- ( ) Social Isolation
- ( ) Substance Misuse
- ( ) Asylum Seeker
- ( ) Carer Support

CognitiveImpairment: (whether person has a cognitive impairment e.g. dementia)

- ( ) No
- (●) Yes
- ( ) Not Available

Risk Result:

```
Inputs:
   sex: Female
   ethnicity: Other
   tenure: Private Tenant
   adleat: No
   adlhyg: Yes
   adltoilet: Yes
   adldress: Yes
   adlhome: No
   careraccess: No
   primarysupport: Mental Health
   cognitiveimpair: Yes
   age: 70
   imd: 46

 Factors:
   age: -0.44765
   imd: -0.419842
   sex: 0.024843
   ethnicity: 0.170159
   tenure: 0.24052
   adleat: 0.537698
   adlhyg: 0
   adltoilet: 0
   adldress: 0
   adlhome: 0.368337
   careraccess: 0.257077
   primarysupport: 1.631076
   cognitiveimpair: 0.210959

 Linear predictor (LP): -1.976823
Predicted risk of care home admission: 0.1217 (12.2%)
```
